# Correlation between tuberculosis-specific interferon-γ release assay and intrathoracic calcification: A cross-sectional study

**DOI:** 10.1371/journal.pone.0270785

**Published:** 2022-07-06

**Authors:** Izumi Yamatani, Kosaku Komiya, Hisayuki Shuto, Marimu Yamanaka, Mari Yamasue, Hiroki Yoshikawa, Kazufumi Hiramatsu, Jun-ichi Kadota

**Affiliations:** Respiratory Medicine and Infectious Diseases, Oita University Faculty of Medicine, Yufu, Oita, Japan; Shandong Public Health Clinical Center: Shandong Provincial Chest Hospital, CHINA

## Abstract

**Background:**

Although persistent tuberculosis (TB) infection is known to cause calcification in the lungs, the relationship between intrathoracic calcification and the results of the interferon-γ release assay (IGRA) has not been fully elucidated. This study aimed to assess the association between intrathoracic calcification and IGRA results.

**Methods:**

We retrospectively included consecutive patients who concurrently underwent chest X-ray, chest computed tomography (CT), and an IGRA. Patients with a current diagnosis of active TB or treatment history of active TB or latent tuberculosis infection (LTBI) were excluded. The association between calcification according to the chest X-ray or CT and IGRA results were analyzed using binomial logistic regression.

**Results:**

This study included 574 patients, and 38 (7%) patients had a positive IGRA result. Patients with a positive result were significantly older and had a higher proportion of comorbidities, and history of tuberculosis exposure compared to those with a negative result. Calcification of the lung field and mediastinal lymph nodes according to chest CT was more frequently observed in patients with a positive IGRA result, whereas no significant difference was observed concerning the proportion of lung field calcification on chest X-ray between patients with positive and negative IGRA results. In multivariate analysis, calcification of mediastinal lymph nodes alone (adjusted odds ratio [OR] = 3.82, 95% confidence interval [CI] = 1.76–8.26) and the combination of lung field and mediastinal lymph node calcification (adjusted OR = 4.12, 95% CI = 1.51–11.76) on chest CT was independently associated with positive IGRA results.

**Conclusions:**

The finding of mediastinal lymph node calcification, with or without lung field calcification, on chest CT was associated with positive IGRA results independent of TB exposure history. Previous TB infection including eliminated TB infection and LTBI can be suspected when calcified lymph nodes in are observed the mediastinum on chest CT.

## Introduction

Although the incidence of tuberculosis (TB) has gradually declined worldwide since approximately 2005 [1], the number of newly reported cases of TB in the elderly population remains high in countries in which the elderly population is increasing [2, 3]. Japan is a TB middle-burden country with a notification rate of 10.1 per 100,000, and patients aged ≥60 years accounted for 72% of all patients with TB in 2020 [4]. Elderly people are at risk for development of secondary tuberculosis due to endogenous reactivation with age-related immunosuppression or increased occasions of immunosuppressant or biological agent usage [5, 6]. To control TB infection in this generation, accurate detection of latent tuberculosis infection (LTBI) is crucial.

Recent research demonstrated a contentious spectrum of bacterial activity and antagonistic immunological responses between LTBI and active TB [7]. However, LTBI is still characterized by evidence of TB infection but no clinical, radiological, or microbiological evidence of active TB disease based on the World Health Organization definition [8]. LTBI is generally diagnosed by tuberculin skin tests or the interferon-γ release assay (IGRA), but the sensitivity of these tests is known to decline in elderly patients [9]. In a study conducted in Japan, although the potential benefits of IGRA were demonstrated in TB contact investigations for elderly people, the positivity rate apparently decreased at more advanced ages [10].

Some guidelines recommend performing chest X-ray regardless of the corresponding IGRA result to screen for LTBI [11] or exclude active TB [8]. Persistent inflammation following initial TB infection may cause calcification in the lung field or mediastinal lymph nodes [12], and this finding might be useful for distinguishing LTBI from active TB [13]. In fact, a systematic review demonstrated that lesions on chest X-ray suggestive of previous TB infection, including calcified lymph nodes, were significantly associated with positive test results for LTBI [14]. However, the determination of whether calcification exists using chest X-ray imaging alone is challenging, and the sensitivity of chest X-ray for previous TB infection was reported to be low at approximately 15% [14].

In recent decades, the opportunities to perform chest computed tomography (CT) have increased with improvements of medical standards and diagnostic imaging. Chest CT has the advantage of detecting calcification with higher accuracy than chest X-ray [15–17]. If the association between LTBI and intrathoracic calcification using chest CT is demonstrated, LTBIs could be suspected using CT features, which can be expected to contribute to improvement in the diagnostic performance of LTBIs. For example, for patients suspected to have false-negative IGRA results and a high risk of future active TB (i.e., receiving aggressive immunosuppression therapy), other signs such as intrathoracic calcification suggestive of LTBI would be informative. Therefore, this study aimed to assess the association between the calcification of lung fields or mediastinal lymph nodes and IGRA results.

## Methods

### Patients and study design

This was a retrospective cross-sectional study conducted at the Department of Respiratory Medicine and Infectious Diseases, Oita University Faculty of Medicine. The 768-bed hospital is located in Oita prefecture, Japan, and its notification rate was 9.5 per 100,000 in 2020 [4]. We included consecutive patients who concurrently underwent chest X-ray, chest CT, and an IGRA from January 2017 to December 2019. This study tolerated time gap within 1 month between taking chest radiological images and the IGRA in accordance with the time interval to conversion of IGRA [18, 19]. In the hospital, physicians may order T-SPOT.TB (Oxford Immunotec Ltd., Abingdon, UK) as an IGRA whenever TB infection is suspected on chest X-ray or CT or screening for TB infection is needed, and this test is covered by universal health insurance. Patients with a current diagnosis of active TB or treatment history of active TB or LTBI were excluded to avoid outcome bias in relation to the identification of calcified lesions and IGRA results. Furthermore, intermediate or indeterminate results of the IGRA were excluded.

The study protocol was approved by the institutional ethics committee of our institution (approval number: 2037; approval date: March 5, 2020), and followed the Declaration of Helsinki Ethical Principles for medical research involving human subjects. The need for informed consent was waived by the committee due to the retrospective design of the study. Information regarding this research was posted at the hospital.

### Data collection

Patient data, including gender, age, body mass index, underlying diseases (e.g., diabetes mellitus, chronic kidney disease with hemodialysis, and immunosuppressive disease), use of immunosuppressant or anti-cancer drugs, past history of TB or dust exposure, laboratory data (white blood cell count, lymphocyte fractionation, albumin level, C-reactive protein level, and lactate dehydrogenase level) were obtained from medical records. The reasons for ordering chest CT by attending physicians were documented if possible.

### Evaluation of chest CT scan findings

A 320-detector row CT scanner (Aquilion ONE, Canon Medical Systems, Tochigi, Japan) was used. Scans were performed using 1.0-mm thick sections of contiguous images from the apex to the lung base. Images were obtained at a window setting of –600 (level) and 1500 (width). If the patient underwent a CT scan before referral to our hospital, the CT scan features were evaluated using the images obtained from the referring institutions. The finding of calcification on chest X-ray (lung field or pleura) or chest CT (lung field, mediastinal lymph node, or pleura) were determined at the mediastinal window. Fibrotic lesions were not assessed in this study because fibrotic change is more frequently observed on chest CT than on chest X-ray in various types of lung diseases including interstitial pneumonia, smoking-related lung diseases, and age-related interstitial changes, and this would be less specific for previous TB infection in elderly people. Chest images were independently evaluated by respiratory medicine specialists (IY and KK) who were blinded to the clinical information. Any disagreement between the presence of these findings in each case was resolved by a review conducted by the same two physicians in order to reach a consensus.

### Statistical analysis

Statistical analyses were performed using the Statistical Package for the Social Sciences software version 22 (IBM Japan, Tokyo, Japan). For two-tailed analyses, 95% confidence intervals were calculated. Interobserver agreement was assessed by kappa value analysis. IGRA positivity was compared by patient characteristics using the chi-squired test. Variables among patients’ backgrounds, laboratory data, chest X-rays, or CT findings with a P value of < 0.05 in the univariate analysis were included in the multivariate analysis. Multivariate analysis was performed for items that showed significant differences in univariate analysis by binomial logistic regression. Eventually, we conducted multivariate analyses in three models because lung field calcification, lymph node calcification, and the combination of both were significantly associated with IGRA positivity in univariate analysis.

## Results

### Baseline characteristics

**Fig 1:**
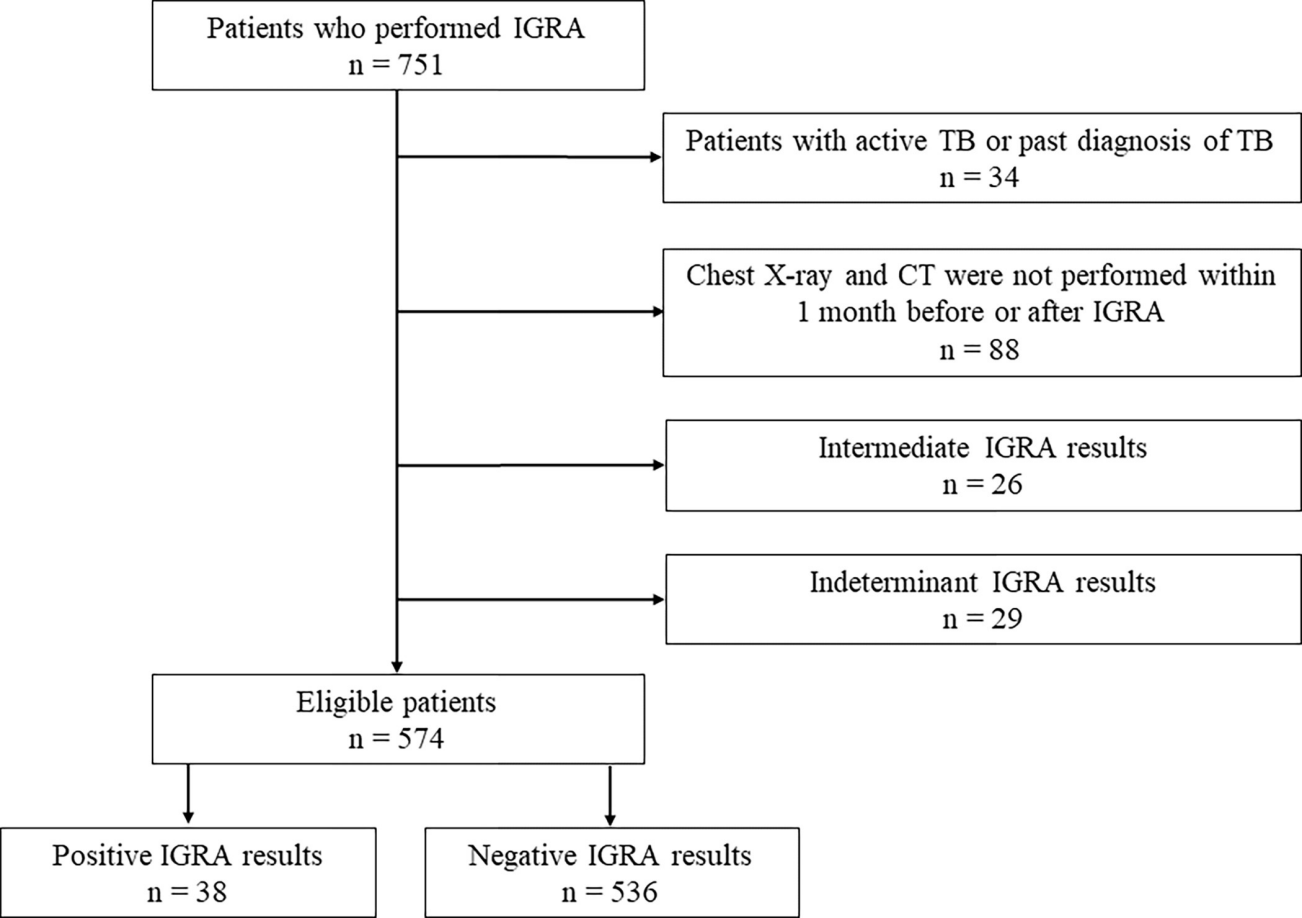
A flow chart of the participants.

In total, 751 patients underwent IGRA during the study period. Of these, four patients were diagnosed with currently active TB, and 30 patients had a past diagnosis of active TB or LTBI. Chest X-ray and chest CT were not concurrently used in 88 patients, and 26 and 29 patients had intermediate or indeterminate IGRA results, respectively. These patients were excluded from this study, as shown in Fig 1. Finally, 574 patients were included, and approximately 50% of the patients were women, and the median patient age was 71 years (interquartile range: 63–77) years.

The main reason for ordering chest CT was further evaluation to determine whether there was an abnormal shadow on chest X-ray for symptomatic patients (n = 294/574, 51.2%) and asymptomatic patients (n = 274/574, 47.7%). Chest CT was also perform for screening before the initiation of immunosuppressant or biological agent therapy (n = 6/574, 1.1%). IGRA was ordered because of the suspicion of LTBI or active TB in all cases, but pretest probabilities by physicians were unclear.

### Prevalence of positive IGRA results

Of the 574 included patients, 38 (7%) had positive IGRA results. Patients with advanced age, those with diabetes mellitus, those who underwent hemodialysis, and those with a history of TB exposure had significantly higher IGRA positivity rates than their counterparts, as presented in Table 1. Lung field calcification was more frequently detected on chest CT (n = 122/574, 21.3%) than on chest X-ray (n = 14/574, 2.4%). The IGRA positivity rate in patients with lung field calcification, pleura, and both according to chest X-ray did not significantly differ from that in their counterparts. By contrast, the positivity rate was significantly higher in patients with lung field calcification, lymph node calcification, and both according to chest CT than in their counterparts.

**Table 1:** IGRA positivity in the baseline characteristics and chest radiological features.

	IGRA positivity		p value
Sex, female / male	16 / 268 (6.0)	22 / 306 (7.2)	0.558
Age (years), > 70 / ≤ 70	25 / 278 (9.0)	13 / 296 (4.4)	0.027
BMI (kg/m^2^), > 22 / ≤ 22	15 / 238 (6.3)	22 / 274 (8.0)	0.452
Use of immunosuppressant, yes / no	2 / 86 (2.3)	36 / 488 (7.4)	0.082
Diabetes mellitus, yes / no	10 / 76 (13.2)	28 / 498 (5.6)	0.014
Hemodialysis, yes / no	3 / 10 (30.0)	35 / 564 (6.2)	0.023
Immunosuppressive disease, yes / no	0 / 8 (0)	38 / 566 (6.7)	1.000
History of dust exposure, yes / no	5 / 79 (6.3)	33 / 495 (6.7)	0.911
History of TB exposure, yes / no	11 / 40 (27.5)	27 / 534 (5.1)	<0.001
WBC (/μL), > 6260 / ≤ 6260	14 / 280 (5.0)	24 / 280 (8.6)	0.093
Ly (%), > 24.05 / ≤ 24.05	20 / 277 (7.2)	18 / 277 (6.5)	0.737
Albumin (g/dL), > 3.90 / ≤ 3.90	13 / 263 (4.9)	24 / 272 (8.8)	0.077
CRP (mg/dL), > 0.19 / ≤ 0.19	18 / 276 (6.5)	19 / 281 (6.8)	0.910
LDH (U/L), > 201 / ≤ 201	13 / 272 (4.8)	24 / 283 (8.5)	0.081
Lung field calcification on X-ray, yes / no	2 / 14 (14.3)	36 / 560 (6.4)	0.236
Pleural calcification on X-ray, yes / no	0 / 5 (0)	38 / 569 (6.7)	1.000
Calcification of both lung field and pleura on X-ray, yes / no	0 / 2 (0)	38 / 572 (6.6)	1.000
Lung field calcification on CT, yes / no	14 / 122 (11.5)	24 / 452 (5.3)	0.015
Lymph node calcification on CT, yes / no	15 / 68 (22.1)	23 / 506 (4.5)	<0.001
Calcification of both lung field and lymph node on CT, yes / no	7 / 25 (28.0)	31 / 549 (5.6)	0.001
Pleural calcification on CT, yes / no	5 / 50 (10.0)	33 / 524 (6.3)	0.364

Data are presented as the number (%). Continuous data was divided using a cutoff as the median.

BMI: body mass index, CI: confidence interval, CT: computed tomography, IGRA: interferon-gamma release assays, LDH: lactate dehydrogenase, TB: tuberculosis, WBC: white blood cell

The kappa values of the radiological findings were as follows: 0.543 for lung field calcification according to chest X-ray, 0.437 for pleural calcification according to chest X-ray, 0.829 for lung field calcification according to chest CT, 0.733 for mediastinal lymph node calcification according to chest CT, and 0.771 for pleural calcification according to chest CT.

### Factors associated with positive IGRA results

Patients with a positive IGRA result were significantly older and had a higher proportion of comorbidities; e.g., diabetes mellitus, hemodialysis, and past history of TB exposure compared to those with negative results (Table 2). Univariate analysis indicated calcifications of lung fields and mediastinal lymph nodes on chest CT were more frequently observed in patients with a positive IGRA result.

**Table 2:** Univariate analysis of the baseline characteristics and chest radiological features associated with positive IGRA results.

	IGRA positive (n = 38)	IGRA negative (n = 536)	odds ratio (95% CI)	p value
Female	16 (42.1)	252 (47.0)	0.82 (0.42–1.60)	0.558
Age (years)	75 (69–81)	70 (61–77)	1.06 (1.02–1.10)	0.001
BMI (kg/m^2^)	21.7 (19.3–24.2)	22.0 (19.6–24.6)	0.98 (0.90–1.06)	0.598
Use of immunosuppressant	2 (5.3)	84 (15.7)	0.30 (0.07–1.27)	0.101
Diabetes mellitus	10 (26.3)	66 (12.3)	2.54 (1.18–5.47)	0.017
Hemodialysis	3 (7.9)	7 (1.3)	6.48 (1.61–26.14)	0.009
Immunosuppressive disease	0 (0)	8 (1.5)	n.a.	n.a.
History of dust exposure	5 (13.2)	74 (13.8)	0.95 (0.36–2.50)	0.911
History of TB exposure	11 (28.9)	29 (5.4)	7.12 (3.22–15.77)	<0.001
WBC (/μL)	5760 (5035–7175)	6225 (4940–8238)	1.01 (0.94–1.08)	0.784
Lymphocyte (%)	24.8 (17.8–28.5)	24.0 (16.3–30.7)	1.01 (0.98–1.04)	0.581
Albumin (g/dL)	3.74 (3.37–4.18)	3.91 (3.53–4.22)	0.79 (0.49–1.29)	0.351
C-reactive protein (mg/dL)	0.18 (0.04–1.09)	0.19 (0.07–1.32)	1.03 (0.96–1.11)	0.428
LDH (U/L)	189 (178–230)	202 (170–234)	1.00 (0.99–1.00)	0.376
Lung field calcification on X-ray	2 (5.3)	12 (2.2)	2.43 (0.52–11.26)	0.258
Pleural calcification on X-ray	0 (0)	5 (0.9)	n.a.	n.a.
Lung field calcification on CT	14 (36.8)	108 (20.1)	2.31 (1.16–4.62)	0.018
Lymph node calcification on CT	15 (39.5)	53 (9.9)	5.94 (2.92–12.08)	<0.001
Calcification of both lung field and lymph node on CT	7 (18.4)	18 (3.4)	6.498 (2.525–16.723)	<0.001
Pleural calcification on CT	5 (13.2)	45 (8.4)	1.65 (0.62–4.44)	0.319

Data are presented as the number (%) or median (interquartile range).

BMI: body mass index, CI: confidence interval, CT: computed tomography, IGRA: interferon-gamma release assays, LDH: lactate dehydrogenase, n.a.: not applicable, TB: tuberculosis, WBC: white blood cell

We conducted multivariate analysis using three models, as presented in Table 3. After adjusting for age, diabetes mellitus, hemodialysis, a history of TB exposure, calcification of mediastinal lymph nodes, and calcification of both of mediastinal lymph nodes and lung fields according to chest CT were independently associated with positive IGRA results, whereas lung field calcification alone was not associated with IGRA positivity. In all models, advanced age, hemodialysis, and a history of TB exposure were commonly associated with positive IGRA results, but diabetes mellitus was not a significant factor.

**Table 3:** Multivariate analysis of the baseline characteristics and chest radiological features associated with positive IGRA results.

	Model 1		Model 2		Model 3	
	odds ratio (95% CI)	p value	odds ratio (95% CI)	p value	odds ratio (95% CI)	p value
Age (years)	1.06 (1.01–1.10)	0.008	1.05 (1.01–1.09)	0.029	1.05 (1.01–1.09)	0.014
Diabetes mellitus	2.27 (0.98–5.28)	0.056	2.21 (0.94–5.18)	0.070	2.33 (1.00–5.43)	0.051
Hemodialysis	7.10 (1.61–31.28)	0.010	7.43 (1.58–34.80)	0.011	7.34 (1.65–32.63)	0.009
History of TB exposure	7.23 (3.11–16.80)	<0.001	6.13 (2.59–14.54)	<0.001	6.77 (2.89–15.88)	<0.001
Lung field calcification on CT	1.99 (0.95–4.15)	0.068				
Lymph node calcification on CT			3.82 (1.76–8.26)	0.001		
Calcification of both lung field and lymph node on CT					4.21 (1.51–11.76)	0.006

CI: confidence interval, CT: computed tomography, IGRA: interferon-gamma release assays, TB: tuberculosis

## Discussion

This study showed that calcification of mediastinal lymph nodes alone or combination of lymph nodes and lung field according to chest CT was associated with positive IGRA results, independently from age, hemodialysis, and past history of TB exposure.

Calcification of lung fields according to chest X-ray was not significantly related to positive IGRA results in the current analysis, which was inconsistent with results in the study showing an association between chest X-ray findings and LTBI [14]. The finding of calcification according to chest X-rays seems to have a limited role in suspecting an LTBI in clinical practice, probably because the detection power of calcification is weak [20]. Furthermore, the interobserver agreement rate for the presence of calcification using chest X-ray turned out to be poor in the current study. This measuring bias might have affected the results. Estimating the probability of previous TB infection based on solely calcification findings according to chest X-ray may be impossible.

Calcification of mediastinal lymph nodes alone or combination with lung field calcification according to chest CT was associated with positive IGRA results in univariate analysis, and interestingly, this was independent of a history of TB exposure. This result indicates that a history of TB exposure alone is insufficient for suspecting previous TB infection. When compared to chest X-ray, chest CT has an advantage in detecting calcification with higher accuracy [16]. In fact, the rate of calcification detection with chest CT was significantly higher than that with chest X-ray, and the interobserver agreement using chest CT was excellent in the current study.

However, it is noted that calcification of mediastinal lymph nodes can be caused by not only previous TB infection but also other persistent inflammation, such as pneumoconiosis [21]. We have documented a history of dust exposure in the past, but we might have insufficiently collected information due to the retrospective nature of the study design. Pathological evaluations of mediastinal lymph nodes with calcification were not conducted in the current study, so the cause and mechanism of calcification remains unclear. Thus, if all cases with calcification of mediastinal lymph nodes are determined to be caused by previous TB infection, then the diagnosis of LTBI or eliminated TB infection would be overestimated.

On the other hand, for patients who were exposed to TB in an early era, calcification of mediastinal lymph nodes would not have been observed. In general, a long-term process would be required for calcification to exist on the persistently inflamed lesion. In the current study, advanced age was significantly related to positive IGRA results in multivariate analysis. Among elderly patients, a longer period would have passed since TB exposure, and this result might explain the requirement for a prolonged time to develop calcification. However, the exact duration required to develop calcification is uncertain. Along with the acquisition of a long interval since TB exposure, the fact that elderly people had more opportunities to be exposed to TB in the high TB burden era would explain the results that both advanced age and calcification were associated with positive IGRA results.

In multivariate analysis, receiving hemodialysis was significantly associated with positive IGRA results, whereas diabetes mellitus was not. While hemodialysis itself is a risk factor for TB infection, in most hemodialysis patients, end-stage renal failure resulted from uncontrolled diabetes mellitus [22]. It is known that well-controlled diabetes does not lead to immunosuppression [23]. Hemodialysis as a factor associated with LTBI might be confounded by uncontrolled diabetes.

As previously stated, the sensitivity of IGRA for detecting previous TB infection may be decreased among elderly people. Thus, lymph node calcification on chest CT, another potential finding suggestive of previous TB infection, would provide useful information concerning the necessity of more careful observation or anti-TB treatment, particularly for patients at high risk of future active TB, such as determining the suitability for aggressive immunosuppression therapy. However, whether a finding of calcification denotes LTBI or prior TB infection eliminated via host immunity without anti-TB drugs remains unknown. Further study is required to determine whether calcification is truly a risk factor for future active TB.

The strength of this study was it was the first to assess the relationship between calcification of lung fields and mediastinal lymph nodes using chest CT and IGRA results on a relatively large scale, considering physicians often perform chest CT scans in Japan. However, several limitations need to be listed. First, inclusion bias may exist because attending physicians determined whether a patient needed performing chest CT and an IGRA based on their assessments. It is unclear whether the physicians suspected LTBI and subsequently performed an IGRA, but almost all patients (n = 568/574, 98.9%) underwent chest CT to further evaluate abnormal chest X-ray findings. Thus, regardless of symptoms, if lymph node calcification is found on chest CT in such situations, it would be a predictive sign for a positive IGRA result. Although calcification on chest X-ray was not associated with IGRA positivity, screening of abnormal findings using chest X-ray might be beneficial. Second, the interobserver’s agreement rate for detection of calcification based on a chest X-ray is apparently low. This is a limitation of the evaluation of calcification using a chest X-ray itself rather than the study design. Third, false positive IGRA results were not able to be assessed. There was no gold standard for diagnosing a LTBI, and it is usually diagnosed based on a positive IGRA result. We previously reported that advanced age and lower lymphocyte levels are mainly associated with false negative IGRA results in a systematic review [9]. In the current study, this information was collected and adjusted in a multivariate analysis. Forth, the relationship between calcification of lymph nodes and IGRA results may be affected by the prevalence rate of TB. In a high TB prevalence area, IGRA positivity would be higher and this might not be associated with increased risk for calcification in a non-aging society. Finally, this study was retrospectively conducted in a single center. Therefore, the results from this study must be carefully generalized.

In conclusion, the finding of mediastinal lymph node calcification, alone or in combination with lung field calcification, on chest CT was associated with positive IGRA results independently of the TB exposure history. The history of TB exposure alone may be insufficient to screen for previous TB infection. Previous TB infection including eliminated TB infection and LTBI can be suspected depending on the prevalence of TB when mediastinal lymph node calcification is observed by chest CT. The background of patients included in this study was heterogeneous. A prospective large study to validate these results in specific populations could encourage increased investigation of previous TB infection and lead to more instances of proper treatment.

## Supporting information

S1 Data (XLSX)

## Abbreviations

CT: computed tomography
IGRA: interferon-γ release assay
LTBI: latent tuberculosis infection
OR: odds ratio
TB: tuberculosis
